# Bridging the Rural Mental Health Gap: Telehealth Delivery of Specialized CBT and MBCT for Veterans With Parkinson's Disease

**DOI:** 10.1111/jrh.70175

**Published:** 2026-06-17

**Authors:** John R. Purcell, Rokas Perskaudas, Rachael B. Miller, Lauren St. Hill, Arlene King, Vibha Reddy, Kait Gilleran, Laura Marsh, Aliya Sarwar, Joel Mack, John E. Duda, Jessica Lehosit, Petty Tineo, Marilyn Hinojosa‐Lindsey, Kristi Ketchum, Solomon Kalkstein, Meredith Shouse, Kayla Maloney, Christopher Liong, Autumn Makin, Gretchen Glenn, Dawn McHale, Susan O'Connor, Alejandro Interian, Roseanne D. Dobkin

**Affiliations:** ^1^ Mental Health and Behavioral Sciences VA New Jersey Healthcare System Lyons New Jersey US; ^2^ Department of Psychiatry Robert Wood Johnson Medical School, Rutgers University Piscataway New Jersey US; ^3^ Mental Health Care Line Parkinson's Disease Research Education and Clinical Center Michael E DeBakey VA Medical Center Houston TX; ^4^ Neurology Care Line Parkinson's Disease Research Education and Clinical Center Michael E DeBakey VA Medical Center Houston Texas US; ^5^ Parkinson’s Disease Research, Education, and Clinical Center VA Portland Healthcare System Portland Oregon US; ^6^ Parkinson's Disease Research, Education and Clinical Center Michael J. Crescenz VA Medical Center Philadelphia Pennsylvania US; ^7^ Parkinson's Disease Research, Education and Clinical Center VA Richmond Healthcare System Richmond Virginia US; ^8^ War Related Illness and Injury Study Center VA New Jersey Healthcare System East Orange New Jersey US

**Keywords:** anxiety, cognitive behavioral therapy, depression, mindfulness

## Abstract

**Purpose:**

The Veteran Affairs (VA) New Jersey Parkinson's Disease (PD) Telepsychotherapy Hub (PD Telepsych Hub) delivers virtual mental health treatments to underserved rural Veterans in partnership with VA Parkinson's Disease Research, Education, and Clinical Centers (PADRECCs). This manuscript outlines the PD Telepsych Hub's hybrid type 2 implementation‐effectiveness framework then presents the clinical and demographic characteristics of Enrolled Veterans along with mental health outcomes for Treatment Engagers receiving individual Cognitive Behavioral Therapy (CBT‐PD) or group Mindfulness‐Based Cognitive Therapy (MBCT‐PD) over the first 5 years of operation (10/2020 to 09/2025).

**Methods:**

Underserved rural Veterans with PD were primarily directly outreached by the PD Telepsych Hub (64%) or referred by VA clinicians. Veterans who met screening criteria and completed a psychiatric consult were enrolled and offered CBT‐PD or MBCT‐PD via telehealth according to preferences and needs. Enrolled Veterans completed baseline questionnaires assessing demographics and health characteristics. Treatment Engagers completed pre‐and‐post measures of mood, anxiety, loneliness, functional change, and treatment satisfaction.

**Results:**

Enrolled Veterans (*N* = 522 across 31 states) were rural (72%), 6% Hispanic, 8% people of color, and on average 71‐years‐old. They reported an average of 7 years since PD diagnosis. Although most met criteria for a mood disorder (86%) and 43% had psychiatric comorbidities, only 9% were receiving psychotherapy at time of program enrollment. CBT‐PD (*n* = 202) significantly reduced depression and anxiety, while MCBT‐PD (*n* = 38) reduced depression. Treatment Engagers (*n* = 240) overall reported high treatment satisfaction (94%).

**Conclusion:**

At baseline, the mismatch between care access and clinical need was striking. Results highlight a growing foundation for real‐world effectiveness in delivering empirically supported, PD‐adapted interventions via telehealth to the highest‐need, lowest‐access populations.

## 1 Introduction

Parkinson's Disease (PD) is one of the most common and debilitating degenerative neurological conditions that impacts the Veteran population [1, 2], with a notable percentage living in rural areas. While defined by the motor triad of tremor, rigidity, and bradykinesia, almost all (90%) Veterans with PD will likely experience debilitating neuropsychiatric symptoms [3]. Depression affects up to 50% of PD patients [3, 4], and is associated with faster physical and cognitive decline [5], poorer quality of life [6], greater caregiver distress [7], disability [8], and increased health care utilization and costs [9]. Depression in Parkinson's disease (dPD) typically does not improve without intervention [10]; effective treatments can help patients regain their previous level of functioning and markedly improve overall PD outcomes [11]. Therefore, depression is a crucial target in overall PD management.

Despite the benefits to treating dPD, it is widely overlooked and sub‐optimally managed in routine clinical practice. In standard PD care, over 60% of patients with significant depressive symptoms are undiagnosed [12, 13]. Recent data from a large cross‐section of the PD community (*N* = 28,310) reflect a comparable shortfall in depression care for Veterans with PD [11]. In this cohort, Veterans reported high rates of current (40%) and untreated (60%) depression. Even among the most severely depressed, 43% were untreated. Rural Veterans face limited access to all forms of PD care, and are at higher risk for the poor outcomes linked to inadequate depression treatment [14].

Addressing dPD requires a specialized, PD‐informed, and integrated multidisciplinary approach that at minimum incorporates neurology (for optimized dopaminergic replacement therapy), psychiatry (for medication management of neuropsychiatric comorbidities), and a mental health professional trained to deliver PD‐adapted psychotherapy [15]. Our specialized cognitive‐behavioral therapy protocol for dPD (CBT‐PD) showed large beneficial effects in two pilot studies (*N* = 21–34) [16, 17] and in three randomized controlled trials (RCTs; *N*s = 72–90) [18, 19, 20] evaluating in‐clinic, telephone, and video‐to‐home modalities in Veteran and community populations. Across all three RCT's, those receiving PD‐informed CBT showed greater improvements in depression, anxiety, and quality of life compared to usual care. Notably, both telemedicine RCTs demonstrated large, durable effects comparable to our in‐clinic RCT, with retention rates exceeding 80% [18, 20]. As personalized care requires more than one evidence‐based approach [21], we also developed a group telehealth Mindfulness‐Based Cognitive Therapy (MBCT) protocol for dPD (MBCT‐PD) that has PD‐specific content, modified structure (e.g., 1.5‐h groups; sequencing of meditations), and other adaptations relevant for chronic neurological conditions (e.g., reinforcement of concrete information and action steps) [22]. MBCT‐PD evidenced feasibility and benefit in pilot work within a clinically depressed/anxious sample [22], building upon previous pilot studies of general MBCT delivered via telehealth [23] and modified MBCT provided in‐person [24, 25] to mostly non‐clinically depressed/anxious PD patients.

However, individuals with PD face a severe shortage of mental health clinicians who understand PD's physical, cognitive, and social challenges, and report that lack of PD‐specific knowledge constitutes a key barrier to treatment utilization and effectiveness [26, 27, 28]. PD‐specific neuropsychiatric phenomena, such as mild cognitive impairment, anxiety, PD stigma, impulse‐control disorders, sleep issues, the fluctuating effects of dopaminergic PD medications, and other PD‐management needs—complicate the course and treatment of depression. Antidepressant medications often constitute first‐line treatment [29], but show mixed efficacy, tolerability, and patient acceptability with many individuals with PD preferring psychotherapy over pharmacological treatment [11, 21, 28, 30], Therefore, delivering accessible, evidence‐based psychotherapy to treat mood symptoms is of paramount importance for the PD population.

Veterans living in rural areas face well‐documented challenges in accessing specialized evidence‐based mental health care [31, 32], and Veterans with PD are especially likely to live in rural areas, placing them at the intersection of two groups already facing significant access barriers [27, 33]. As a result, rural Veterans with PD are particularly vulnerable to the compounded effects of geographic isolation, PD‐related mobility and transportation difficulties, and the overall shortage of mental health services in rural communities. These factors contribute to lower engagement in care and higher burden of mental illness, despite equal or greater need [34].

The Veteran Affairs (VA) New Jersey PD Telepsych Hub (PD Telepsych Hub) was established in 2020 using an integrated hub‐and‐spoke model [35] to—in collaboration with the VA Parkinson's Research, Education, and Clinical Centers (PADRECCs) [1]—deliver PD‐informed depression treatment directly into the homes of rural Veterans with PD. Given that preliminary pilot studies and multiple subsequent RCTs support the efficacy of CBT‐PD for treating dPD across various modalities (e.g., in‐person, telephone, video) and populations (e.g., general, Veteran) [16, 17, 18, 19, 20], the PD Telepsych Hub adopted a hybrid type 2 implementation‐effectiveness framework [36, 37] for the first 5 years of operation with relatively equal continuous focus on (1) increasing direct engagement of rural Veterans with PD and (2) evaluating the effectiveness of the empirically supported interventions offered by the Hub as it expands. While CBT‐PD is the Hub's primary treatment option, MBCT‐PD was developed and pilot‐tested to meet an emerging need expressed by our Veterans for efficacious complementary group treatments and was subsequently integrated into Hub operations as well as continuous evaluation of effectiveness. The present manuscript briefly outlines the operational model of the PD Telepsych Hub, presents engagement indicators (e.g., general outreach metrics, attitudes about care, treatment attendance, satisfaction with program), and describes the clinical/demographic characteristics of Enrolled Veterans, in addition to pre–post change in the mental health (e.g., depression, anxiety, loneliness) of Veterans who received PD‐informed individual CBT‐PD or group MBCT‐PD.

## 2 Methods

All data analyzed in this project and presented here in the aggregate were collected for operational and quality improvement purposes as part of an Office of Rural Health Innovative Program Grant, and were approved as operational (non‐research) by the VHA. Targeted outreach, referrals, and subsequent treatment began in October 2020 and remain active; all available data up to 09/2025 were included in analyses.

### 2.1 Outreach and Enrollment

**FIGURE 1:**
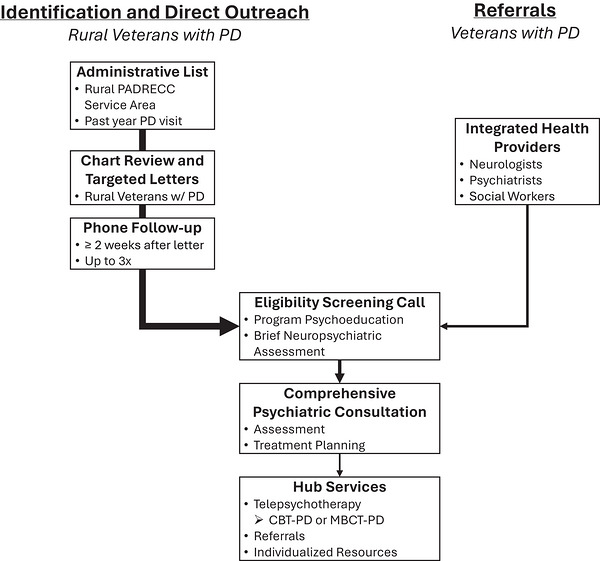
Veterans Affairs (VA) New Jersey Parkinson's Disease (PD) Telepsychotherapy Hub (PD Telepsych Hub) outreach, referral, and enrollment workflow. CBT‐PD, Parkinson's Disease‐informed Cognitive Behavioral Therapy; MBCT‐PD, Parkinson's Disease‐informed Mindfulness‐Based Cognitive Therapy; PADRECC, Veterans Affairs Parkinson's Disease Research, Education, and Clinical Center.

The PD Telepsych Hub has two enrollment pipelines: (1) primarily direct outreach to rural Veterans with PD, and (2) referral from a VA provider to the PD Telepsych Hub regardless of rurality (Figure 1). Direct outreach utilizes a regularly updated health care operations administrative list to identify Veterans with a rural ZIP code (Rural Urban Commuting Area Code^1^ ≥2 [primary] and/or ≥1.2 [secondary]) [38, 39, 40] and a PD‐related visit in the past year within PADRECC service areas for VA medical chart review. Early iterations of the PD Telepsych Hub targeted rural Veterans with PD in New Jersey (*n* = 58 of Enrolled Veterans) prior to formal partnerships with PADRECCs. As the PD Telepsych Hub forms partnerships with PADRECCs (Houston, 03/2021; Portland, 01/2022; Philadelphia, 06/2023; Richmond, 11/2023), Hub access via referral becomes immediately available across each PADRECC's multi‐state service area (see Diaz and Bronstein [1]; colored map available online [41]) while direct outreach and engagement expands state‐by‐state prioritized by rurality and remains ongoing. Veterans are identified (e.g., confirmed PD diagnosis in medical chart and residence in a rural zip code) to receive a letter about the program with scheduled phone follow‐up to provide additional information and address questions. Interested Veterans then undergo brief neuropsychiatric screening by phone along with psychoeducation about the program, followed by a 60‐ to 90‐min psychiatric consult with a Hub clinician for formal enrollment.

The PD Telepsych Hub follows an inclusive enrollment policy. It requires only a confirmed PD diagnosis in the VA medical record, demonstrated mental health treatment needs (e.g., severity of anxiety/depression, presence of diagnosable disorder(s), self‐reported need) to enroll and receive diagnostic consultation, and the ability to engage in and benefit from telehealth psychotherapy (e.g., supports for digital barriers, adequate cognitive functioning, manageable medication‐related perceptual disturbances). Restated, while direct outreach efforts by the PD Telepsych Hub focus on rural Veterans with PD and significant mental health concerns, services are always accessible to a broader population of Veterans with PD to maximize reach and impact.

### 2.2 Procedure

Following the psychiatric consult, which incorporates screening information, chart review, interview, and diagnosis, the evaluating clinicians develop personalized, PD‐informed mental health treatment plans in collaboration with other Hub clinicians or supervisors, and additional collateral information is gathered as needed. Primary intervention options offered by the PD Telepsych Hub include individual CBT‐PD or group MBCT‐PD telehealth‐to‐home psychotherapies. Supplementary services are available and frequently provided regardless of direct treatment provision by the PD Telepsych Hub, including referral to PD‐specialized integrated health services (e.g., psychiatrist, neurologist, PT/OT, speech therapist) and individualized resources (e.g., self‐guided CBT‐PD manual, local support services, curated digital resource lists specific to the Veteran). Veterans are invited to participate in both CBT‐PD and MBCT‐PD based on clinical need, though not concurrently. Treatment recommendations and assignments are primarily based on Veteran preference for individual versus group modality, with final assignment dictated by clinical judgment regarding appropriateness and timing; no random treatment assignment is utilized. Veterans with highest clinical need (e.g., severity of symptoms, acute distress, increased suicide risk) are prioritized for individual CBT‐PD given it involves more direct and quicker intervention compared to potentially waiting for a MBCT‐PD group cohort to form, and currently has well‐demonstrated efficacy [16, 17, 18, 19, 20]. The individual and group psychotherapies offered by the PD Telepsych Hub are delivered via VA Video Connect (VVC), a secure VA‐maintained video‐to‐home platform, with telephone used as a supplemental or backup connection modality. Outreach, recruitment, data collection, and screening are conducted by trained PD Telepsych Hub staff; diagnostic intake, treatment planning, and treatment delivery are provided or directly supervised by licensed mental health professionals with CBT‐PD and/or MBCT‐PD experience.

Veterans who participated in a psychiatric consult, which includes treatment recommendations and planning are referred henceforth as “Enrolled Veterans.” Among Enrolled Veterans, some did not participate in telehealth psychotherapy for a variety of reasons (e.g., referring provider requested one‐time consult for treatment optimization, low mental health symptomology, dementia, conflict with other treatment, preference for self‐help resources and/or local referrals, unknown reasons) and are referred to as “One‐Time Consults.” Enrolled Veterans that engaged in at least one telehealth psychotherapy session of any modality are broadly considered “Treatment Engagers” broken down into “CBT‐PD Engagers” and “MBCT‐PD Engagers” accordingly.

### 2.3 Interventions

The primary telehealth psychotherapy provided by the PD Telepsych Hub is CBT‐PD, structured as a 10‐session (1‐h each) treatment program with an accompanying self‐guided manual [16, 17, 18, 19, 20]. CBT‐PD emphasizes psychoeducation and behavioral activation (BA) throughout, followed by thought‐logging, cognitive restructuring, stress/anxiety management, relaxation training, sleep hygiene, and relapse prevention. Its sequencing, content, and practices are also adapted for PD populations with unique psychosocial challenges, physical disability, and neuropsychiatric comorbidities [42]; for further CBT‐PD details, see Dobkin et al. [19, 20] and for meta‐analyses on related approaches to CBT‐PD, see Alnajjar [43], Yu et al. [44], and Wu et al. [45]. The PD Telepsych Hub also adopts a Chronic Care Model [46] approach by individually tailoring clinical delivery and content to fluctuating clinical status while maintaining core CBT‐PD components. This includes adjusting session length and frequency, mixing modalities (e.g., short phone check‐ins between sessions for behavioral engagement), active care partner involvement, and content modifications based on individual needs. Examples of modifications include flexible at‐home practice completion formats (e.g., video/audio vs. written, digital aids, care support), modified module sequencing (e.g., earlier sleep/anxiety management), incorporation of supplemental modules (e.g., CBT for Chronic Pain, mindfulness, safety planning), and content simplification (e.g., BA emphasis with simplified restructuring, content repetition, more in‐session guided practice). The PD Telepsych Hub has also developed a formal two‐day CBT‐PD training course, which launched in 2023 to provide workforce development, training psychologists, social workers, nurse practitioners, graduate students, and post docs. This training course establishes competent foundations in CBT‐PD and emphasizes appropriate modifications based on individual clinical needs while adhering to core therapeutic mechanisms such as behavioral activation (BA) [47].

Group MBCT‐PD begins with a 1‐h individual session focused on psychoeducation and an introduction to mindfulness practice, followed by eight 90‐min cohort model group sessions delivered by VVC [22]. MBCT‐PD incorporates meditation and experiential exercises to cultivate nonjudgmental awareness of habitual, negative reactions (e.g., hopelessness, avoidance) to PD symptoms (e.g., tremor). Session topics include present‐focused meditations, mindful breathing, body scanning, thought/emotion awareness, acceptance, and relapse prevention. By developing mindful acceptance of PD symptoms, participants increase their capacity to manage PD challenges with awareness and adaptive coping. For further details see Interian et al. [22].

Following active treatment completion, Veterans are offered optional post‐active treatment booster sessions (individual for CBT‐PD, monthly groups for MBCT‐PD) to help maintain treatment gains and prevent relapse. Post‐treatment data reported in the current manuscript were collected after active treatment but before booster participation; no follow‐up clinical data were collected or analyzed after post‐treatment program evaluation.

### 2.4 Measures and Assessment Procedure

During phone screening, Veterans are administered the Montreal Cognitive Assessment‐Blind (MoCA‐Blind; an adapted version of the MoCA administered without visual stimuli) [48, 49], Geriatric Depression Scale (GDS‐15) [50], and Generalized Anxiety Disorder‐7 (GAD‐7) [51]. Enrolled Veterans are sent links to a VA survey (e.g., Qualtrics) assessing demographics, PD and mental health history, health care utilization, beliefs on PD‐specific mental health services, and symptom severity using the Beck Depression Inventory (BDI‐II) [52], Patient Health Questionnaire‐9 (PHQ‐9) [53], Parkinson's Anxiety Scale (PAS) [54], and UCLA 3‐Item Loneliness Scale (3LS) [55]. As the underreporting of MH symptoms on self‐report measures can serve as a treatment referral barrier for some Veterans [56, 57], Enrolled Veterans are outreached via telephone if a self‐reported BDI‐II score is <16 and/or symptomatology reported during intake starkly contrasts symptomatology endorsed on self‐report assessments and BDI scores are collaboratively reviewed and updated accordingly. Additionally, indications of potentially heightened suicide risk captured by the BDI‐II (item 9) and PHQ‐9 (item 9) are discussed by PD Telepsych Hub clinicians and addressed according to VA policy. Therapy Engagers are sent a post‐treatment VA Qualtrics survey reassessing the BDI‐II, PHQ‐9, and 3LS along with a unidirectional, patient‐rated version of the Patient Global Impression of Change Scale (PGICS) [58] to gauge overall perception of significant functional improvement and most items from the 8‐item Client Satisfaction Questionnaire (CSQ‐8) [59] to gauge treatment satisfaction. All collected self‐report data are for program evaluation purposes, so while Enrolled Veterans are encouraged to provide complete data, items are not “forced response” and Veterans are not excluded from receiving hub services due to incomplete data.

### 2.5 Data Curation and Analyses

Missing and conflicting demographic or clinical data at baseline were resolved via VA chart review wherever possible. Analyses utilized all available data depending on focus (i.e., all Enrolled Veterans, Treatment Engagers with complete data; detailed missingness is reported in Table A1). Further data cleaning and analyses were performed using Rstudio (R v.4.5.1). Large Language Models (e.g., Microsoft Co‐Pilot) were used to troubleshoot lines of code and calculate percentiles at the group level. No individual participant data was uploaded to an LLM. Individual missing data points for otherwise completed symptom measures (*n* = 8 across all 522 participants) were imputed by rounding the average score for the total of each instrument. Pre‐ and post‐treatment symptom changes were analyzed using linear mixed‐effect models fit by restricted maximum likelihood (REML) with Satterwaite's method T‐tests. Modeling random effects at the level of each participant accounted for individual differences for the pre‐treatment scores while time point (i.e., pre/post treatment) was modeled as a fixed effect. The standard effect size was calculated by multiplying the unstandardized fixed effect by the ratio of the standard deviations of the symptom scores at each time point. Between‐subject variability and within‐subject variance were calculated as respective measures of score variation due to individual differences and residual error.

## 3 Results

**FIGURE 2:**
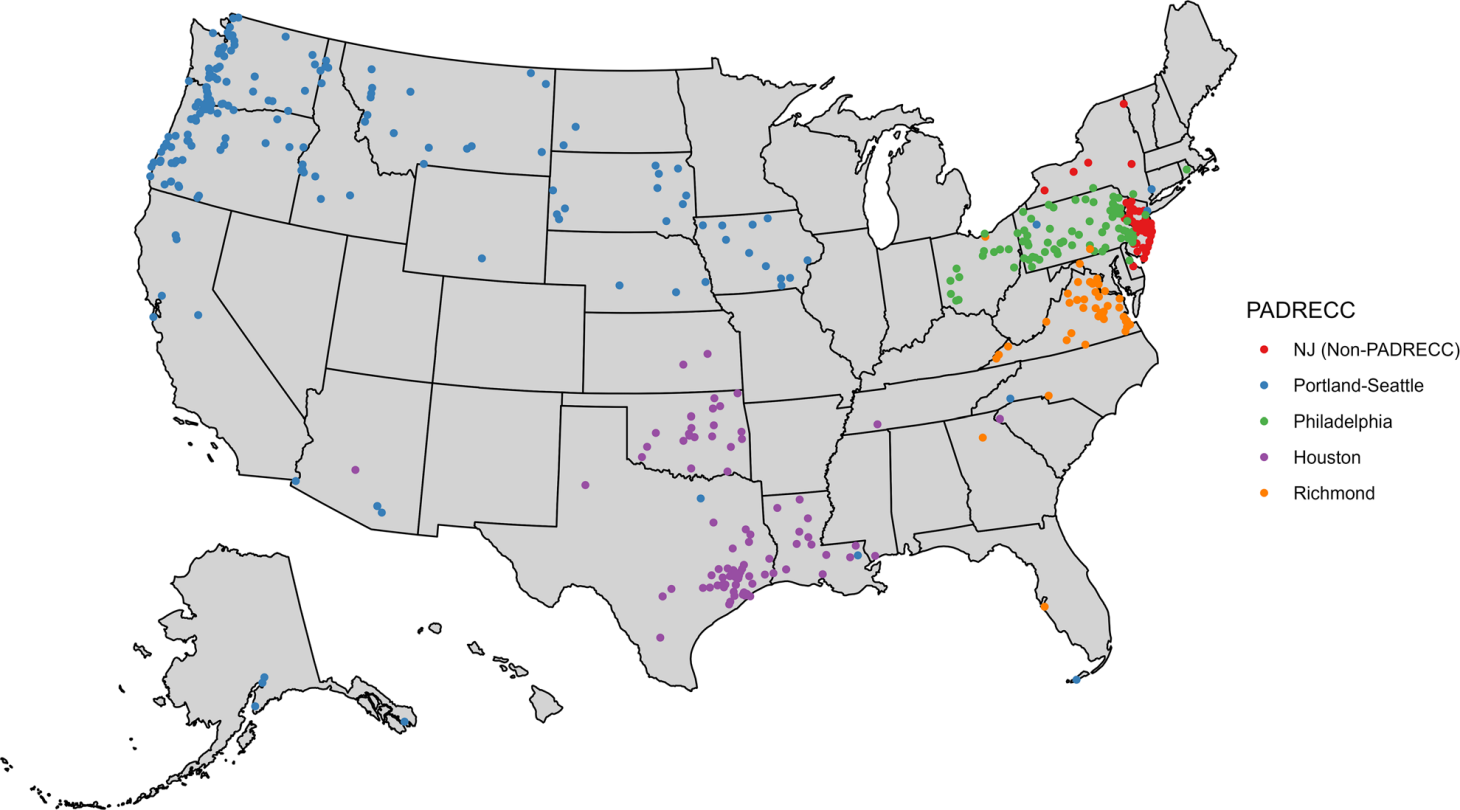
Geographic Map of Enrolled Veterans (n=522) by PADRECC. PADRECC, Veterans Affairs Parkinson's Disease Research, Education, and Clinical Center.

**FIGURE 3:**
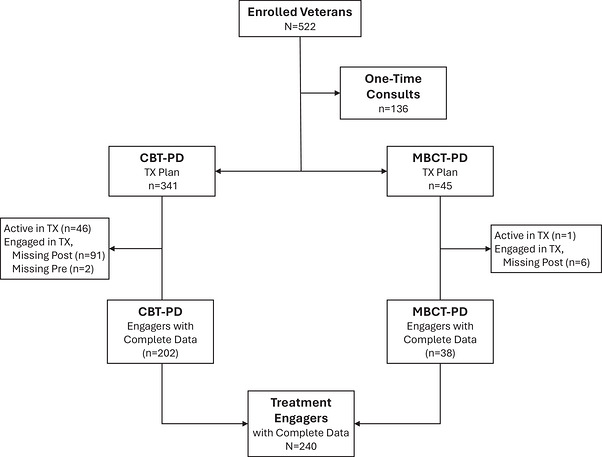
Enrolled Veteran (*N* = 522) flowchart. CBT‐PD, Parkinson's Disease‐informed Cognitive Behavioral Therapy; MBCT‐PD, Parkinson's Disease‐informed Mindfulness‐Based Cognitive Therapy; TX, treatment.

Over the first 5 years of operation (10/2020 to 09/2025), the PD Telepsych Hub has chart‐reviewed over 5000 unique rural Veterans with PD and directly reached out (letter and up to 3 follow‐up phone calls) to 1982 rural Veterans with PD across at least 22 states^2^ regarding Hub services. A total of 522 unique Veterans across 31 states^3^ enrolled in the Hub (see Figure 2 for a map of the geographic reach of our program), with 64.4% (*n* = 336) identified via the direct outreach and contact methods described above, and 33.9% (*n* = 177) referred by a PADRECC or other VA clinician (1.7% other sources: webinar, support group). Of the 522 Veterans, 136 completed a One‐Time Consult, 47 were actively engaged in therapy within the Hub (at the time of data cutoff), 240 had engaged in therapy and completed pre‐ and post‐intervention measures (Treatment Engagers with complete data), and 99 engaged in therapy but did not complete both pre‐ and post‐intervention measures (Treatment Engagers with partial data). See Figure 3 for an Enrolled Veteran flowchart.

### 3.1 Demographic and Clinical Characteristics

Full details are provided in Tables 1 and 2. Enrolled Veterans were primarily older adults (*M*_age_ = 71.25) with mean 7.13 years since PD diagnosis. They were predominantly male (95.8%) and self‐identified as White (91.7%); 6.0% reported Hispanic ethnicity. The majority (80.17%) reported either being married or living as married with a significant partner, and most Veterans had a high school education or higher (97.2%). Over 72% of Veterans resided in a rural area, living on average 57.74 driving miles away from their geographically nearest VA Medical Center and 264.68 driving miles away from their geographically nearest PADRECC. On a 1–10 (low‐high) scale, Veterans reported average overall health (*M* = 5.50), low awareness of local mental health resources (*M* = 4.14), moderately high belief in the benefits of psychotherapy for the treatment of mental health concerns in PD (*M* = 6.88), and a strong belief that providers should have sufficient knowledge of PD when providing psychotherapy for this medical population (*M* = 8.61).

**TABLE 1:** Sample demographics.

	Enrolled Veterans (*n* = 522)	Treatment Engagers (*n* = 240)	CBT‐PD Engagers (*n* = 202)	MBCT‐PD Engagers (*n* = 38)
**Rurality** ^a^				
Rural zip code	72.99%	71.17%	72.28%	68.42%
**Driving distance to closest VA institutions** ^b^				
Miles to VA Medical Center	57.74 (54.12)	57.83 (48.59)	58.31(45.06)	55.26 (64.90)
Miles to PADRECC	264.68 (344.97)	233.85 (303.61)	237.42 (309.02)	214.85 (276.03)
**Age**	71.24 (8.43)	70.78 (8.25)	71.06 (8.18)	69.31 (8.60)
**Male sex at birth** ^c^	500 (95.79%)	229 (95.42%)	194 (96.03%)	35 (92.10%)
**Race**				
Asian	1 (0.21%)	0	0	0
Black	18 (3.73%)	6 (2.50%)	6 (2.97%)	0
Pacific Islander	1 (0.21%)	1 (0.42%)	1 (0.50%)	0
Native American	6 (1.24%)	4 (1.67%)	4 (1.98%)	0
Other^d^	11 (2.28%)	4 (1.67%)	1 (0.50%)	3 (7.89%)
White	442 (91.70%)	225 (93.75%)	190 (94.06%)	35 (92.11%)
Prefer not to answer	3 (0.62%)	0%	0%	0%
**Ethnicity**				
Hispanic	29 (6.01%)	13 (5.42%)	9 (4.46%)	4 (10.53%)
Prefer not to answer	9 (1.87%)	3 (1.25%)	3 (1.49%)	0
**Relationship status**				
Divorced	56 (11.62%)	30 (12.50%)	26 (12.87%)	4 (10.53%)
Living as married	18 (3.73%)	9 (3.75%)	7 (3.47%)	2 (5.26%)
Married	368 (76.35%)	181 (75.42%)	151 (74.75%)	30 (78.95%)
Never married	9 (1.87%)	5 (2.08%)	5 (2.48%)	0
Separated	4 (0.83%)	2 (0.83%)	1 (0.50%)	1 (2.63%)
Widowed	25 (5.19%)	13 (5.42%)	12 (5.94%)	1 (2.63%)
Prefer not to answer	2 (0.41%)	0	0	0
**Educational attainment**				
8th grade	3 (0.63%)	0	0	0
Some high school	8 (1.67%)	3 (1.26%)	3 (1.49%)	0
High school/GED	94 (19.67%)	47 (19.67%)	45 (22.28%)	2 (5.41%)
Some college	164 (34.31%)	84 (35.15%)	67 (33.17%)	17 (45.95%)
College	149 (31.17%)	75 (31.38%)	62 (30.69%)	13 (35.14%)
Graduate school	58 (12.13%)	30 (12.55%)	25 (12.38%)	5 (13.51%)
Prefer not to answer	2 (0.42%)	0	0	0

*Note*: Treatment Engagers are subsumed under Enrolled Veterans, and only Treatment Engagers with complete (pre–post) data are reported.

Abbreviations: CBT‐PD, Parkinson's Disease‐informed Cognitive Behavioral Therapy; MBCT‐PD, Parkinson's Disease‐informed Mindfulness‐Based Cognitive Therapy; VA, Veterans Affairs; PADRECC, Veterans Affairs Parkinson's Disease Research, Education, and Clinical Center.

^a^ Rural Urban Commuting Area (RUCA) Codes based on 2010 Census data were utilized for rurality designation due to the 2020 Census being in‐process at the time of PD Telepsych Hub formation. A cutoff RUCA code of ≥2 (primary)/1.2 (secondary) was set per Office of Rural Health recommendations for maximum inclusivity. Efforts were also made to incorporate additional state‐level indicators of rurality to account for delay in release of 2020 Census data.

^b^ Calculated using the shortest Google Maps drive path from each Enrolled Veterans's zip code to all VA Medical Centers (*N* = 172) and PADRECCS (*n* = 6) then identifying nearest VA Medical Center/PADRECC.

^c^ One participant identified as non‐binary, and one identified as a transgender woman.

^d^ Write‐ins included Hispanic/Spanish (*n* = 4), Native American and White (*n* = 2).

**TABLE 2:** Clinical characteristics.

	Enrolled Veterans (*N* = 522)	Treatment Engagers (*n* = 240)	CBT‐PD Engagers (*n* = 202)	MBCT‐PD Engagers (*n* = 38)
**PD**				
PD duration	7.13 (5.34)	6.47 (4.95)	6.69 (5.06)	5.33 (4.14)
PD age of onset	64.04 (9.67)	64.25 (9.26)	64.30 (9.26)	63.99 (9.38)
**MH history**				
Age of first MH concern	58.75 (16.02)	58.66 (17.15)	60.60 (15.01)	53.27 (19.20)
Age of most recent MH concern	68.58 (10.79)	68.52 (10.48)	68.95 (10.56)	65.81 (9.74)
**Primary psychiatric (DSM‐5) diagnosis**				
Mood disorder^a^	449 (86.02%)	205 (85.42%)	176 (87.13%)	29 (76.32%)
Anxiety disorder^b^	41 (7.85%)	22 (9.17%)	16 (7.92%)	6 (15.79%)
Other^c^	29 (5.56%)	13 (5.42%)	10 (4.95%)	3 (7.89%)
None	3 (0.57%)	0	0	0
**Number of diagnoses**				
Single diagnosis	297 (56.90%)	132 (55.00%)	107 (52.97%)	25 (65.79%)
Two or more diagnoses	222 (42.53%)	108 (45.00%)	95 (47.03%)	13 (34.21%)
**Treatment at intake** ^d^				
Current antidepressant medication	276 (52.87%)	126 (52.50%)	105 (51.98%)	21 (55.26%)
Current psychotherapy	47 (9.00%)	21 (8.75%)	16 (7.92%)	5 (13.16%)
**MH treatment history** ^e^				
MH meds	41 (8.69%)	40 (16.88%)	38 (19.10%)	2 (5.26%)
MH talk therapy	80 (16.95%)	26 (10.97%)	18 (9.05%)	8 (21.05%)
MH meds/talk therapy	141 (29.87%)	72 (30.38%)	60 (30.15%)	12 (31.58%)
**Primary PD provider in last year**				
Primary care physician	33 (6.98%)	18 (7.56%)	18 (9.00%)	0 (0%)
Neurologist	410 (86.68%)	206 (86.55	169 (84.50%)	37 (97.37%)
Did not meet with physician	31 (6.55%)	14 (5.88%)	13 (6.50%)	1 (2.63%)
Number of past year PD Provider^f^ visits	2.78 (2.23)	2.66 (1.75)	2.65 (1.81)	2.68 (1.38)
**Engagement with other PD care in last year** ^g^				
Occupational therapist	119 (25.11%)	60 (25.21%)	51 (25.50%)	9 (23.68%)
Physical therapist	261 (55.06%)	141 (59.24%)	117 (58.50%)	24 (63.16%)
Speech therapist	112 (23.63%)	55 (23.11%)	49 (24.50%)	6 (15.79%)
PD support group	70 (14.77%)	42 (17.65%)	32 (16.00%)	10 (26.32%)
PD exercise class/group	97 (20.46%)	54 (22.69%)	47 (23.50%)	7 (18.42%)
Other	78 (16.46%)	43 (18.07%)	34 (17.00%)	9 (23.68%)
None	110 (23.21%)	40 (16.81%)	36 (18.00%)	4 (10.53%)
**Clinical and cognitive characteristics at phone screening**				
GDS‐15	7.38 (3.45)	7.31 (3.41)	7.55 (3.37)	5.97 (3.35)
GAD‐7	9.70 (5.22)	9.67 (5.25)	9.95 (5.16)	8.16 (5.51)
MoCA‐Blind	17.33 (2.65)	17.91 (2.49)	17.80 (2.60)	18.54 (1.73)
**Beliefs on MH services for those with PD and overall health (range 1–10)**				
Awareness of local MH in PD service availability	4.14 (2.91)	4.22 (2.95)	4.29 (2.86)	3.86 (3.40)
Importance of PD understanding in MH providers	8.61 (1.96)	8.64 (2.00)	8.66 (1.94)	8.54 (2.30)
Usefulness of MH therapy for those with PD	6.88 (2.48)	7.07 (2.48)	7.11 (2.46)	6.81 (2.61)
Overall health	5.50 (1.84)	5.51 (1.81)	5.52 (1.84)	5.43 (1.72)
**Treatment session attendance**				
Sessions attended^h^	—	—	10.70 (2.03)	6.58 (1.23)

*Note*: For missing data, see Table A1. For histogram of sessions attended, see Figure 4. Treatment Engagers are subsumed under Enrolled Veterans, and only Treatment Engagers with complete (pre–post) data are reported.

Abbreviations: CBT‐PD, Parkinson's Disease‐informed Cognitive Behavioral Therapy; MBCT‐PD, Parkinson's Disease‐informed Mindfulness‐Based Cognitive Therapy; MH, mental health; PD, Parkinson's disease.

^a^ Primarily major depressive disorder but also included persistent depressive disorder, other/unspecified depression, and bipolar disorder.

^b^ Primarily generalized anxiety disorder but also included other/unspecified anxiety disorder and specific phobias.

^c^ Other disorders included posttraumatic stress disorder, other/unspecified trauma or stressor‐related disorder, and adjustment disorder.

^d^ Antidepressant medication and psychotherapy at intake was determined via medical chart review. Any indication of prescribed use of an antidepressant regardless of purpose was counted while psychotherapy was defined as at least once a month visits with a mental health professional primarily focused on some form (e.g., individual, group, etc.) of structured psychotherapy (e.g., not adjunctive to primary medication management, neurology, or an unstructured support group, exercise/relaxation class, or related service).

^e^ Caution is warranted in interpreting lifetime mental health history as survey questions did not provide definitions of “mental health medications” as well as “talk therapy” so Veterans appeared to variably but consistently miscategorize (e.g., false positives, false negatives) both past mental health medications and talk therapies (e.g., endorsed talk therapy experience that was primarily medication management, disregarded structured group psychotherapy, etc.) based on subsequent medical chart review and psychodiagnostic intake.

^f^ Primary care physician or neurologist.

^g^ Options were select‐all‐that‐apply or otherwise leave blank so percentages will not sum to 100%.

^h^ Individual sessions for CBT‐PD, group sessions for MBCT‐PD (not including 1 individual session prior to group initiation).

At phone screening, Veterans reported mild‐to‐moderate levels of depressive (*M*_GDS‐15_ = 7.38) and anxiety symptoms (*M*_GAD‐7_ = 9.70), and mild‐to‐moderate cognitive impairment (*M*_MoCA‐Blind_ = 17.33). The majority of Enrolled Veterans were diagnosed with a mood disorder (86.0%) or anxiety disorder (7.5%) as a primary diagnosis (3% other, 2% trauma disorder, <1% Adjustment/None) following psychodiagnostic intake interview. Almost half (42.5%) met criteria for multiple DSM‐5 disorders with an anxiety (47.8%) or trauma/stressor disorder (31.5%) being the most frequent secondary diagnoses; a trauma/stressor disorder was the most common (46.7%) among those with a third DSM‐5 diagnoses (*n* = 15). The average age of first self‐reported mental health concern was 58.75, and the average age of most recent MH concern was 68.58. At the point of enrollment, 52.9% were taking antidepressant medications, and only 9.0% were receiving psychotherapy (approximately 15% reported lifetime history of standalone talk therapy of unknown structure/content).

### 3.2 Treatment Outcome Data

Overall, telehealth treatment provided by the PD Telepsych Hub (i.e., combined CBT‐PD and MBCT‐PD) resulted in significant reductions in depression (see Table A2 and Figure A1: BDI‐II, *M*_Pre_ = 21.06, *M*_Post_ = 13.73, *β* = –0.79; PHQ‐9, *M*_Pre_ = 10.88, *M*_Post_ = 8.00, *β* = –0.51), anxiety (PAS, *M*_Pre_ = 19.58, *M*_Post_ = 14.34, *β* = –0.56), and loneliness (3LS, *M*_Pre_ = 5.45, *M*_Post_ = 5.07, *β* = –0.22). The majority of Treatment Engagers (67.9%) with complete data reported significant functional improvement on the PGICS (≥5; at least “*Moderately better, and a slight but noticeable difference*”). Treatment satisfaction was consistently high across interventions with 94.9% reporting high satisfaction with services and 96.6% noting they would refer a friend. See Table 4 for treatment satisfaction responses for all Treatment Engagers.

**FIGURE 4:**
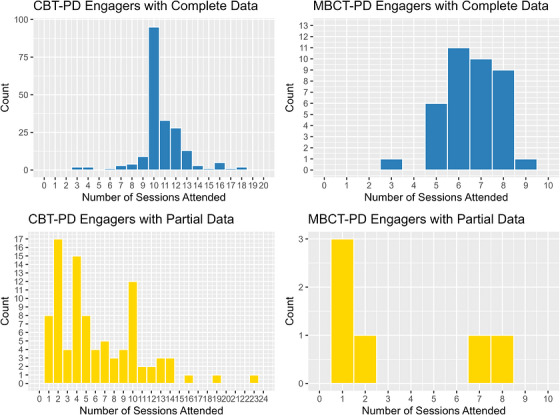
Histograms of therapy sessions attended by Treatment Engagers with complete and partial data. Partial data = having self‐report measures from either before or after treatment, but not both timepoints. CBT‐PD, Parkinson's Disease‐informed Cognitive Behavioral Therapy; MBCT‐PD, Parkinson's Disease‐informed Mindfulness‐Based Cognitive Therapy.

**FIGURE 5:**
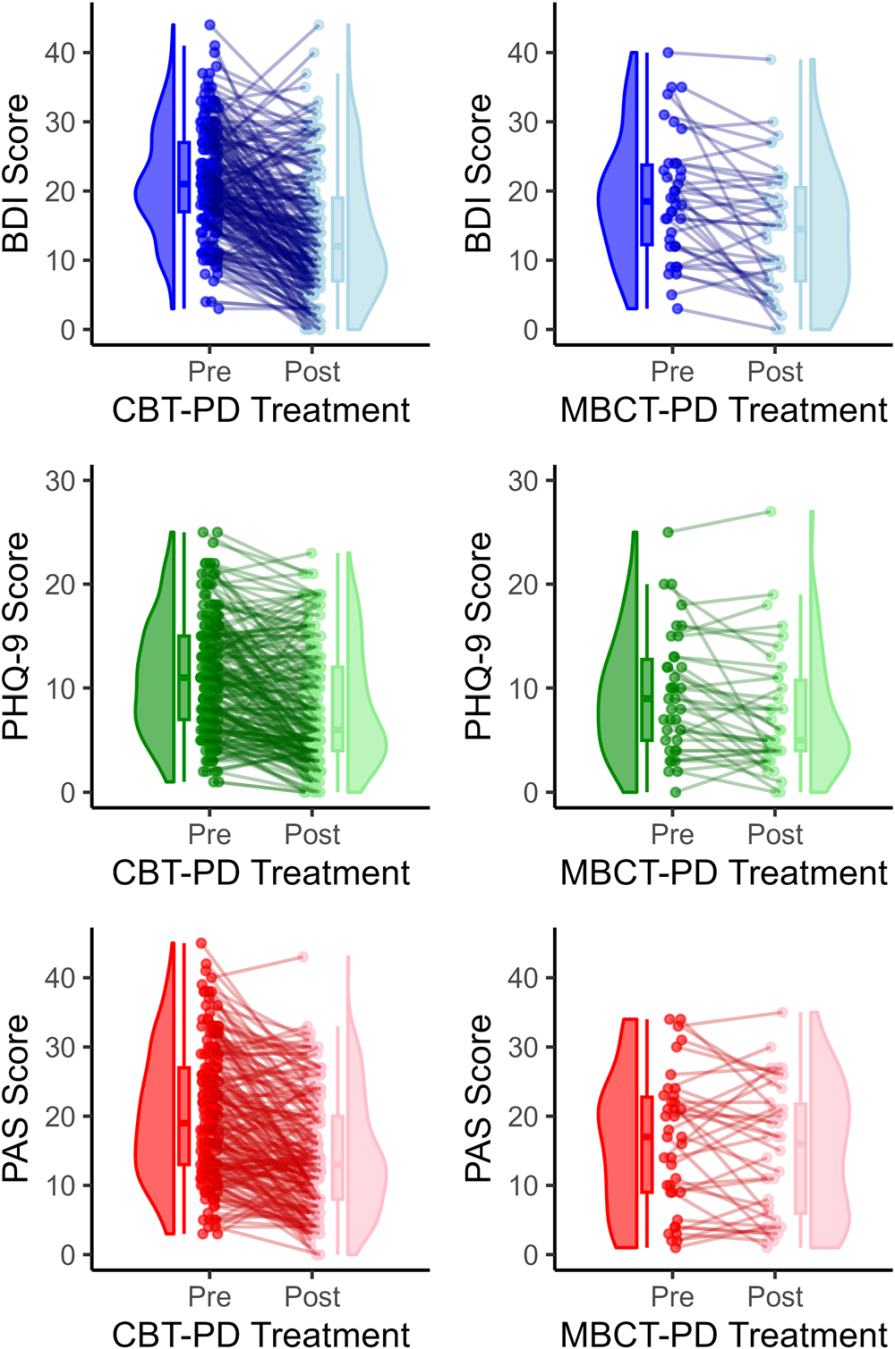
Pre‐ and post‐treatment depression and anxiety scores for CBT‐PD and MBCT‐PD engagers with complete data. Enrolled Veteran (*N* = 522) flowchart. BDI, Beck Depression Inventory II; CBT‐PD, Parkinson's Disease‐informed Cognitive Behavioral Therapy; MBCT‐PD, Parkinson's Disease‐informed Mindfulness‐Based Cognitive Therapy; PAS, Parkinson Anxiety Scale; PHQ‐9 Patient Health Questionnaire.

Session attendance for all Treatment Engagers with complete and partial data by treatment (CBT‐PD/MBCT‐PD) is provided in Figure 4. CBT‐PD Engagers with complete data attended, on average, 10.70 individual therapy sessions (in the acute treatment period). CBT‐PD resulted in significant reductions in BDI‐II,^4^ PHQ‐9, PAS, and 3LS scores (Table 3, Figure 5). Most Veterans (71.0%) reported functionally significant improvement on the PGICS, and high treatment satisfaction (96.5%) with CBT‐PD (Table 4).

**TABLE 3:** Pre‐ and post‐treatment outcomes analyses for CBT‐PD and MBCT‐PD engagers with complete data.

CBT‐PD (*n* = 202)	Pre mean (SD)	Post mean (SD)	CBT‐PD statistic	Standardized effect size [95% CI]	Between‐subject variance (SD) and residual variance (SD)
**Beck Depression Inventory (BDI) Total**	21.43 (7.75)	13.65 (8.97)	*b* = –7.78, *SE* = 0.61, *t*(201) = –12.80, *p* < 0.001	*β* = –0.84 [–0.97, –0.71]	*σ* ^2^ = 33.00 (5.74) *σ* ^2^ = 37.28 (6.11)
**Patient Health Questionnaire (PHQ‐9) Total**	11.15 (5.38)	8.10 (5.53)	*b* = –3.05, SE = 0.34, *t*(198) = –8.99, *p* < 0.001	*β* = –0.54 [–0.66, –0.42]	*σ* ^2^ = 18.28 (4.28) *σ* ^2^ = 11.47 (3.39)
**Parkinson's Anxiety Scale (PAS) Total**	20.21 (9.18)	14.22 (8.31)	*b* = –5.99, SE = 0.55, *t*(198) = –10.97, *p* < 0.001	*β* = –0.65 [–0.76, –0.53]	*σ* ^2^ = 47.05 (6.86) *σ* ^2^ = 29.68 (5.45)
**PAS‐Persistence Subscale**	10.62 (4.24)	7.58 (4.24)	*b* = –3.04, SE = 0.26, *t*(198) = –11.51, *p* < 0.001	*β* = –0.68 [–0.79, –0.56]	*σ* ^2^ = 11.04 (3.32) *σ* ^2^ = 6.94 (2.64)
**PAS‐Episodic Subscale**	5.17 (3.40)	3.61 (2.82)	*b* = –1.55, SE = 0.22, *t*(198) = –7.10, *p* < 0.001	*β* = –0.48 [–0.62, –0.35]	*σ* ^2^ = 5.00 (2.24) *σ* ^2^ = 4.76 (2.18)
**PAS‐Avoidant Subscale**	4.42 (3.00)	3.03 (2.47)	*b* = –1.40, SE = 0.20, *t*(198) = –7.00, *p* < 0.001	*β* = –0.49 [–0.63, –0.35]	*σ* ^2^ = 3.60 (1.90) *σ* ^2^ = 3.96 (1.99)
**UCLA 3‐Item Loneliness Scale (3LS) Total**	5.54 (1.75)	5.13 (1.59)	*b* = –0.41, SE = 0.14, *t*(115) = –2.88, *p* = 0.005	*β* = –0.25 [–0.42, –0.08]	*σ* ^2^ = 1.59 (1.26) *σ* ^2^ = 1.20 (1.10)

MCBT‐PD (*n* = 38)	Pre‐MBCT	Post‐MBCT	MBCT‐PD statistic	Standardized effect size [95% CI]	Between‐subject variance (SD) and residual variance (SD)
**Beck Depression Inventory (BDI) Total**	19.13 (8.87)	14.18 (9.19)	*b* = –4.95, SE = 1.18, *t*(37) = –4.21, *p* < 0.001	*β* = –0.53 [–0.78, –0.28]	*σ* ^2^ = 55.33 (7.44) *σ* ^2^ = 26.27 (5.13)
**Patient Health Questionnaire (PHQ‐9) Total**	9.47 (5.77)	7.47 (5.89)	*b* = –2.00, SE = 0.64, *t*(37) = –3.14, *p* = 0.003	*β* = –0.34 [–0.56, –0.12]	*σ* ^2^ = 26.28 (5.13) *σ* ^2^ = 7.73 (2.78)
**Parkinson's Anxiety Scale (PAS) Total**	16. 32 (9.71)	15.00 (9.48)	*b* = –1.32, SE = 1.07, *t*(37) = –1.23, *p* = 0.225	*β* = –0.14 [–0.36, 0.08]	*σ* ^2^ = 70.49 (8.40) *σ* ^2^ = 21.60 (4.65)
**PAS‐Persistence Subscale**	8.39 (4.99)	7.71 (4.38)	*b* = –0.68, SE = 0.66, *t*(37) = –1.04, *p* = 0.305	*β* = –0.15 [–0.43, 0.13]	*σ* ^2^ = 13.82 (3.72) *σ* ^2^ = 8.22 (2.87)
**PAS‐Episodic Subscale**	4.11 (3.22)	3.82 (3.07)	*b* = –0.29, SE = 0.35, *t*(37) = –0.84, *p* = 0.407	*β* = –0.09 [–0.31, 0.13]	*σ* ^2^ = 7.64 (2.76) *σ* ^2^ = 2.27 (1.51)
**PAS‐Avoidant Subscale**	3.82 (2.99)	3.47 (2.78)	*b* = –0.34, SE = 0.37, *t*(37) = –0.93, *p* = 0.356	*β* = –0.12 [–0.37, 0.13]	*σ* ^2^ = 5.79 (2.41) *σ* ^2^ = 2.55 (1.60)
**UCLA 3‐Item Loneliness Scale (3LS) Total**	4.95 (1.53)	4.82 (1.79)	*b* = –0.14, SE = 0.39, *t*(21) = –0.35, *p* = 0.727	*β* = –0.08 [–0.56, 0.39]	*σ* ^2^ = 1.13 (1.07) *σ* ^2^ = 1.63 (1.28)

*Note*: Linear mixed‐effects model of pre–post symptom scores with random effects for each subject.

**TABLE 4:** Satisfaction with therapy for Treatment Engagers.

	Treatment Engagers (*N* = 236)	CBT‐PD Engagers (*n* = 198)	MBCT‐PD Engagers (*n* = 38)
Did you get the kind of service you wanted?	94.92%	96.46%	86.84%
To what extent has our program met your needs?	85.60%	88.38%	71.05%
If a friend was in need of similar help, would you recommend our program to him or her?	96.61%	96.46%	97.37%
How satisfied are you with the amount of help you have received?	94.92%	94.44%	97.37%
Have the services you received helped you to deal more effectively with your problems?	93.64%	94.44%	89.47%
If you were to seek help again, would you come back to our program?	94.07%	95.45%	86.84%

*Note*: Percent of high score on self‐report. A high score is an endorsement of 3 or 4, while a low score is 1 or 2.

MBCT‐PD Engagers with complete data attended a mean of 6.58 group therapy sessions with all (97.4%) except one Veteran completing an individual introduction to MBCT‐PD (i.e., due to scheduling difficulties and new group cohort starting imminently). MBCT‐PD resulted in significant reductions in BDI‐II^4^ and PHQ‐9 scores (Table 3, Figure 5). Veterans did not report significant reductions in PAS and 3LS scores. Half of MBCT‐PD Engagers (52.9%) endorsed functionally significant improvement on the PGICS, and moderately high treatment satisfaction (86.6%) with MBCT‐PD (Table 4).

## 4 Discussion

The Veterans Affairs New Jersey Parkinson's Disease Telepsychotherapy Hub (PD Telepsych Hub) fills a long‐standing treatment gap in mental health care using PD‐informed, evidence‐based psychotherapies delivered via telehealth to primarily rural Veterans. The program achieves this goal through flexibility and innovations, such as a telehealth hub model, direct‐to‐home engagement, and an expanding network of regional partners to maximize reach while maintaining high levels of clinical effectiveness. Enrolled Veterans who engaged in CBT‐PD demonstrated significant decreases in depression and anxiety. Specifically, the decrease in depression was comparable to two previous RCT trials comparing CBT‐PD against TAU in community [18] and Veteran samples [20]. Similar effect sizes were also found in an in‐person RCT comparing CBT‐PD to clinical monitoring [19], and two small pilot studies of CBT‐PD via telephone [17] and telehealth modalities [16]. Enrolled Veterans who engaged in MBCT‐PD likewise demonstrated significant decreases in depression, which complements previous pilot studies of in‐person [25] and telehealth MBCT for PD patients [23], and expands upon our own previous pilot study of MBCT‐PD [22]. Clinical outcomes from the present investigation are notable considering that the CBT‐PD sample (*N* = 202) is larger than the total treated sample of all efficacy studies of CBT‐PD to‐date (*N* = 178), and the MBCT‐PD sample (*N* = 38) is larger than previous pilot studies (*N*s = 16–27) [22, 23, 25]. It is similarly notable that effectiveness was demonstrated within the complex clinical context of Enrolled Veterans (neuropsychiatric comorbidity; history of chronic and recurrent depressive episodes, which for many predated onset of PD motor symptoms).

At baseline, there was a potent mismatch between care access and clinical need in this population. Both Enrolled Veterans and Treatment Engagers demonstrated high clinical need via: (1) diagnosable DSM‐5 disorder(s); (2) significant clinical symptoms; and (3) self‐reported importance of and a need for PD‐adapted mental health care. Several indices reflected low access to quality mental health care: (1) 73% were rural; (2) only 9% were receiving psychotherapy (a 91% treatment gap); and (3) self‐reported low access to care. At their time of enrollment, Treatment Engagers who were not rural and/or were receiving antidepressants also presented with symptoms of untreated or undertreated depression, and all Enrolled Veterans demonstrated a variety of general, intersecting, and specific barriers to treatment. Ultimately, however, Treatment Engagers consistently reported high rates of treatment satisfaction across all queried indicators (e.g., needs met, quality of service, likelihood of referral) following participation. As such, these results highlight a growing foundation for real‐world effectiveness in delivering empirically supported, PD‐adapted CBT treatment via telehealth to the highest‐need, lowest‐access populations.

The paramount strengths of the PD Telepsych Hub that support effectiveness are innovation through an integrated telehealth hub model [35], multiple direct engagement pathways, and flexibility in clinical delivery using the Chronic Care Model to maximize engagement and impact [46]. Most Enrolled Veterans connected to the PD Telepsych Hub in response to direct outreach and engagement efforts (direct‐to‐home mailing and phone follow‐up) while a third were referred to the PD Telepsych Hub in the context of an established VA provider relationship. Both approaches are likely to decrease stigma and increase motivation for mental health treatment by providing psychoeducation about neuropsychiatric concerns common in PD, increasing awareness of available services for those concerns, and maintaining multiple ways for interested Veterans to virtually engage with the PD Telepsych Hub according to their needs (e.g., individualized PD resources, mental health consultation, self‐help mental health resources, choice of active treatment modalities). Moreover, the integration of clinicians trained by the PD Telepsych Hub in CBT‐PD as direct service providers underscores the feasibility of maintaining high levels of efficacy while also bypassing treatment barriers by using an integrated hub model. Our hub has trained 61 clinicians across the nation to‐date since 2023, and approximately three quarters of the PD Telepsych Hub's clinical encounters were facilitated by providers who have completed our specialized 2‐day training in PD mental health.

Several notable features also illustrate the client‐tailored provision of empirically supported treatments offered by the PD Telepsych Hub. Regarding clinical delivery, the average MoCA‐Blind scores of Enrolled Veterans and Treatment Engagers indicates successful treatment for people with some degree of cognitive impairment, which is often a clinical reality of PD, significant barrier to treatment, and potentially exclusionary criterion for more stringent treatment programs. Relatedly, the number of sessions completed by CBT‐PD participants featured increased variability toward the upper range, reflecting facilitation of additional sessions based on individual clinical need (e.g., inclusion of care partners, repetition, shorter/more frequent sessions, neurological/physical changes). Finally, although follow‐up data were not captured for analysis, every Treatment Engager was offered follow‐up booster sessions after the active portion of treatment; Veterans were also welcome to participate in both individual CBT and group MBCT as long as they were not concurrent. The PD Telepsych Hub hence remains an active, flexible, and continuing point of engagement to mental health services for all Veterans with PD.

Our findings provide insights for engaging rural Veterans, therapy tailored for specific medical populations, and telehealth treatment provision more broadly. While the PD Telepsych Hub operates similarly to other established VA programs in using a hub‐and‐spoke model and integrating telehealth [60, 61], it is the only program that provides specialized, evidence‐based psychotherapy to rural Veterans with PD as well as unique in directly outreaching every potentially eligible Veteran within PADRECC service areas while still allowing standard referral procedures. Targeted identification, direct outreach via multiple methods, and utilization of telepsychotherapy can effectively reduce rural mental health care gaps while maintaining high levels of treatment efficacy and adapting to emerging needs. Moreover, regularly training remote providers to administer empirically supported adaptations and operating as a fully virtual hub (compared to hybrid in‐clinic/virtual) allows for quicker expansion across geographic areas as well as integration into wider health care systems operations. Summarized, effective implementation of telehealth rural mental health programs can be improved by: (1) increasing engagement via direct outreach to target populations in addition to building partnerships with health care providers and community partners for referrals [28]; (2) training providers in the nuances of the population served to better tailor individualized treatment [20]; (3) fostering infrastructure for, adoption of, and competency with telehealth devices/software [62]; and (4) integrating treatment programs with health care systems, self‐management strategies, and other community resources [35, 46]. Many of these recommendations reflect the growing focus on integrated PD‐informed telehealth care outside of the VA [63], and policy initiatives focused on improving telehealth (e.g., pay parity for mental health services and interstate licensing compacts such as IMCL and PSYPACT) support increased telehealth utilization and help mitigate barriers to implementing similar care models in the wider community [64].

Although the primary purpose of the PD Telepsych Hub is to deliver quality mental health treatment effectively rather than compare relative efficacies, Veterans receiving individual CBT‐PD appeared to have higher symptom scores at intake and reported greater benefits (e.g., significant changes in more variables, higher effect size of changes) along with somewhat higher treatment satisfaction rates than group MBCT‐PD participants. This discrepancy may be partly related to the PD Telepsych Hub policy of connecting Enrolled Veterans with the highest clinical need to individual psychotherapy. This explanation is supported by lower mean levels of depression and anxiety at baseline for MBCT‐PD participants compared to individual CBT‐PD participants. Furthermore, it is pertinent to highlight that MBCT‐PD was developed, pilot‐tested, and integrated into the PD Telepsych Hub during active program expansion to meet an emerging need expressed by rural Veterans with PD for complementary treatment options (e.g., group mindfulness‐based PD‐informed telepsychotherapy) with our results supporting the feasibility of full telehealth delivery of MBCT‐PD while maintaining significant impact on depression in a hard‐to‐reach, underserved population usually lacking access to even one type of PD‐informed psychotherapy. This is often the reality of real‐world implementation, and integrating MBCT‐PD into operations during live program implementation, growth, and adaptation constitutes a point of strength for our Hub.

MBCT‐PD also currently undergoes ongoing modifications based on real‐time implementation efforts and subsequent insights. For example, MBCT‐PD groups were organized and facilitated specifically for female Veterans with PD and for Veterans with earlier onset PD following self‐advocacy by Enrolled Veterans and internal analysis revealing an opportunity to meet emerging needs. Additionally, following facilitation of 18 MBCT‐PD group cohorts by the PD Telepsych Hub to‐date, group facilitators have informally observed a pattern that Veterans with PD who have higher baseline cognitive functioning, some past experience with individual CBT treatment, and more chronicity to their depression (versus recent first onset) may have a higher likelihood of beneficial outcomes from MBCT‐PD participation, which reflects meta‐analyses and reviews of MBCT for depression in general populations [65]. Future studies should explore MBCT‐PD efficacy in greater depth across contexts as the PD Telepsych Hub continues to implement groups, adapt accordingly, and collect more program evaluation data.

## 5 Limitations

This study had several limitations. First, not all Enrolled Veterans provided complete data across all variables and time points (see Table A1 for full missingness details). Furthermore, some data elements and collection methods changed with PD Telepsych Hub development, expansion, and/or administrative influence (e.g., VA policy requirements), which introduced systematic missingness for variables added after Hub establishment on 10/2020 such as the PHQ‐9/PAS (added 02/2021), PGICS (06/2022), and 3LS (03/2023). However, overall missingness was relatively low across study subsamples and there was almost no missing data for the primary depression variable (BDI‐II) in analyses. Second, there was also a clear overrepresentation of White males in PD Telepsych Hub enrollees. This is partly attributable to the general demographics of the aging Veteran population and a higher prevalence of PD among males versus females [66] but nevertheless limits generalizability to more gender, racially, and ethnically diverse populations. Third, while Veterans are encouraged to use video conferencing whenever possible, and are provided ample support via VA Information and Technology Services (e.g., VA‐tablet, personal device setup, technical troubleshooting), telephone delivery of individual CBT‐PD was utilized as backup to maintain continuity of care and was not tracked. Although a recent meta‐analysis indicates differing efficacy between phone‐ and video‐delivered psychotherapy among Veterans [67], previous pilot and RCT studies of CBT‐PD via telephone [17, 18] and video modalities [16, 20] suggest comparable effect sizes regardless of delivery modality.

Finally, flexibility in clinical delivery presents a tradeoff between fidelity to a manualized treatment protocol and meeting the immediate clinical needs of the population served by the PD Telepsych Hub. It is possible that variations made to increase engagement and personalize treatment to individual needs dilute active mechanisms of the manualized treatment and/or introduce confounding mechanisms. This tension inherently exists when implementing any empirically supported mental health treatments in the “real world,” which prompted the PD Telepsych Hub to develop a formal CBT‐PD training that reviews adapting treatment facets to individual patient needs with greater competence. Additional data elements (e.g., engagement with other VA services, sleep quality, perceived social support) that the PD Telepsych Hub is considering capturing as it expands, along with refinement of our hybrid type 2 implementation effectiveness framework and targets for greater specificity [36, 37, 68], may also help mitigate confounds between efficacy, effectiveness, and implementation.

## 6 Conclusion

The PD Telepsych Hub is an integrated, virtual hub‐and‐spoke network effectively delivering efficacious telepsychotherapy services to underserved rural Veterans with PD since 2020. Prior to engagement with our program, less than 15% of Enrolled Veterans had previously received evidence‐based psychotherapy as a primary intervention for depression or anxiety, despite great clinical need. Overall, results support the Hub's hybrid type 2 implementation‐effectiveness focus on targeted direct outreach alongside building referral networks, and maintaining clinical effectiveness of telehealth treatment while adapting to emerging needs. At its current expansion rate to one new PADRECC service area per year, the PD Telepsych Hub will soon become a fully integrated national program (i.e., formally partnered with all 6 PADRECCs and directly outreaching within their geographic areas). The PD Telepsych Hub will simultaneously continue to collect a variety of established as well as novel data points to track effectiveness with regularly updated primary analyses. Future supplemental analyses under consideration include but are not limited to effect of treatment sequencing of MBCT‐PD following CBT‐PD (current *N* = 36), treatment response prediction at baseline, and exploring various moderators/mediators of treatment engagement as well as benefit. The PD Telepsych Hub is likely to maintain effectiveness while expanding its overall reach and impact on rural Veterans with PD into the future.

## Conflicts of Interest

All authors have reviewed and approved the final version of this manuscript. The authors declare no conflicts of interest.

## Supporting information



SupportingInfomation: jrh70175‐sup‐0001‐SuppMat.docx

## Endnotes

1 Rural Urban Commuting Area (RUCA) Codes based on 2010 Census data were utilized for rurality designation due to the 2020 Census being in‐process at the time of PD Telepsych Hub formation. A cutoff RUCA code of ≥2 (primary)/1.2 (secondary) was set per Office of Rural Health recommendations for maximum inclusivity. Efforts were made to incorporate additional state‐level indicators of rurality to account for delay in release of 2020 Census data.

2 States (*n* = 22) represented in targeted mailings include (alphabetically): AK, FL, IA, ID, LA, MD, MS, MT, ND, NE, NJ, OH, OK, OR, PA, SD, TX, VA, VT, WA, WV, WY.

^3^
States (*n* = 31) represented by Enrolled Veterans (alphabetically): AK, AZ, CA, CT, DE, FL, GA, IA, ID, KA, LA, MS, MT, NC, ND, NE, NJ, NY, OH, OK, OR, PA, RI, SC, SD, TN, TX, VA, WA, WV, WY.

4 All analyses utilizing the BDI‐II were also re‐analyzed using non‐reviewed pre‐treatment scores (*n* = 83 [16%] of Enrolled Veterans; *n* = 38 [19%] of CBT‐PD Engagers; *n* = 12 [32%] of MBCT‐PD Engagers). Reviewed/non‐reviewed BDI‐II total score means were relatively similar for CBT‐PD (*M*_Reviewed_ = 21.43 [SD = 7.75], *M*_Non‐reviewed_ = 20.36 [8.47]) with more discrepancy for MBCT‐PD (*M*_Reviewed_ = 19.13 [8.87], *M*_Non‐reviewed_ = 17.84 [9.97]), likely due to the smaller sample size. Main results remained significant using non‐reviewed pre‐treatment BDI‐II scores for CBT‐PD (*b* = –6.71, SE = 0.61, *t*(201) = –10.99, *p* < 0.00, *β* = –0.72 [–0.85, –0.59]) and MBCT‐PD (*b* = –3.66, SE = 1.20, *t*(37) = –3.05, *p* = 0.004, *β* = –0.38 [–0.62, –0.13]), therefore only analyses utilizing reviewed scores are reported.
