# Supplementary material for: Bridging the Rural Mental Health Gap: Telehealth Delivery of Specialized CBT and MBCT for Veterans With Parkinson's Disease
Source: J Rural Health. 2026 Jun 17;42(2):e70175. doi: 10.1111/jrh.70175 (PMC13276434; doi:10.1111/jrh.70175)
Supplement: Supplementary file 1 — SupportingInfomation: jrh70175‐sup‐0001‐SuppMat.docx [file JRH-42-0-s001.docx]

**Supplementary Materials**

**Title:** Bridging the Rural Mental Health Gap: Telehealth Delivery of Specialized CBT and MBCT for Veterans with Parkinson's Disease.

**Authors:** Purcell, J.R., Perskaudas, R., Miller, R.B., St. Hill, L., King, A., Reddy, V., Gilleran, K., Marsh, L., Sarwar, A., Mack, J., Duda, J.E., Lehosit, J., Tineo, P., Hinojosa-Lindsey, M., Ketchum, K., Kalkstein, S., Shouse, M., Maloney, K., Liong, C., Makin, A., Glenn, G., McHale, D., O’Connor, S., Interian, A., Dobkin, R.D.

**Table A1. Missing Data or Data Not Endorsed for all Variables**

|  | Enrolled Veterans (N=522) | Treatment Engagers (n=240) | CBT-PD Engagers (n=202) | MBCT-PD Engagers (n=38) |
| --- | --- | --- | --- | --- |
| Age^a^ | None | None | None | None |
| % Male Sex at Birth^a^ | None | None | None | None |
| Race | 40 (7.7%) | None | None | None |
| Hispanic Ethnicity | 40 (7.7%) | None | None | None |
| Relationship | 40 (7.7%) | None | None | None |
| Education | 44 (8.4%) | 1 (0.4%) | None | 1 (2.6%) |
| Primary Diagnosis^b^ (Mood/Anxiety/Other/None) | None | None | None | None |
| # of Diagnoses^b^ (Single/Multiple) | None | None | None | None |
| # Using Antidepressant Medication at Intake (Y/N)^b^ | None | None | None | None |
| # In Psychotherapy at Intake (Y/N)^b^ | None | None | None | None |
| % Rural^c^ | None | None | None | None |
| Mean Distance to VA^c^ | None | None | None | None |
| Mean Distance to PADRECC^c^ | None | None | None | None |
| PD Duration | 44 (8.4%) | 2 (0.8%) | 2 (1.0%) | None |
| **Past Year PD-related Visit** (**Y/N**) | 48 (9.2%) | 2 (0.8%) | 2 (1.0%) | None |
| **↳Y**: *# of PD-Related Physician Visits in Last Year^d^* | 66 (12.6%) | 8 (3.3%) | 7 (3.5%) | 1 (2.6%) |
| **↳Y**: *Primary Provider seen in Last Year^d^* | 49 (9.4%) | 2 (0.8%) | 2 (1.0%) | None |
| **↳Y/N**:*Other Past Year PD-Related Care^d^* | 48 (9.2%) | 2 (0.8%) | 2 (1.0%) | None |
| **MH Concern Since PD Dx** (**Y/N**) | 48 (8.4%) | 3 (1.3%) | 3 (1.5%) | None |
| **↳Y**: *Age of First MH Concern^e^* | 64 (12.3%) | 11 (4.6%) | 9 (4.5%) | 2 (5.3%) |
| **↳Y**: *Age of Most Recent MH^e^ Concern* | 63 (12.1%) | 7 (2.9%) | 5 (2.5%) | 2 (5.3%) |
| **↳Y**: *MH Treatment History^e^* | 50 (9.6%) | 3 (1.3%) | 3 (1.5%) | None |
| GDS at Screening^f^ | 5 (1.0%) | None | None | 1 (2.6%) |
| GAD-7 at Screening^f^ | 5 (1.0%) | None | None | 1 (2.6%) |
| MOCA at Screening^f^ | 9 (1.7%) | None | None | 1 (2.6%) |
| # of Individual Sessions^g^ | N/A | N/A | None | None |
| # of Group Sessions^g^ | N/A | N/A | N/A | None |
| BDI | 49 (9.4%) | None | None | None |
| PHQ-9^h^ | 52 (10.0%) | 3 (1.3%) | 3 (1.5%) | None |
| PAS^h^ | 52 (10.0%) | 3 (1.3%) | 3 (1.5%) | None |
| PGICS^i^ | N/A | 44 (18.3%) | 40 (19.8%) | 4 (10.5%) |
| 3LS^j^ | 220 (42.1%) | 102 (42.5%) | 86 (42.6%) | 16 (42.1%) |

Note. Treatment Engagers are subsumed under Enrolled Veterans, and only Treatment Engagers with complete (pre-post) data are reported. “None” is used here to represent 0 (0.0%) but is presented textually for easier visual identification of where missingness is occurring.

a. Missing Age and Birth Sex were resolved by using age/sex information available at consultation from the VHA EHR.

b. Diagnoses, use of any anti-depressant, and current psychotherapy at consultation were obtained through systematic chart review of standard intake consultation reports in the VHA EHR for each Veteran. Diagnoses were drawn from all listed diagnoses in order written under the “Diagnoses” section present in most VHA intake reporting per policy requirements. Current antidepressant use/psychotherapy statuses at enrollment were also primarily drawn from intake reports. Missing or unclear statuses were resolved by further reviewing Veteran’s chart for all documented visits for any possible VHA or community involvement in structured psychotherapy (e.g., at least monthly individual or group psychotherapy that is not primarily supportive in nature) and any active antidepressant medications in the VHA EHR within a month period before/after date of consultation.

c. Accurate address information was readily available for all Veterans at time of enrollment as it is crucial for direct outreach to rural Veterans who may otherwise not be connected through other modalities as well as receiving hard-copies of all treatment-related materials if they proceeded with telehealth psychotherapy through the hub. Hence, any geographically derived data reported/presented (e.g., rurality, mean distances) were not subject to missingness.

d. Follow up questions regarding PD-care frequency and providers were only presented to Veterans who reported any past year PD-related care visit (**↳Y**), except for other PY PD-related care, which was presented to all Veterans regardless of past year primary PD-care (**↳Y/N**).

e. Follow up questions regarding mental health history and treatment were only presented to Veterans that reported any mental health concern since PD diagnosis (**↳Y**).

f. Screening measures are fully administered and entered by trained hub staff which generally limits missingness except for a few cases reported here that reflect some Veterans enrolling during the beginning phase of the program when screenings/contact first occurred prior to program inception, and some Veterans that presented as unique referrals from another source (e.g., non-associated VHA program) for specialized consultation.

g. Encounter information is regularly tracked for program evaluation and reporting purposes, so it is fully available for all Veterans that engage with the Hub in any form through internal tracking as well as VHA EHR documentation. However, session attendance is only applicable to those Veterans that engaged in any treatment with individual session attendance being applicable to both CBT/MBCT modalities and group session attendance only to MBCT.

h. Added 02/2021.

i. Added 06/2022; assessed at post only.

j. Added 03/2023.

**Table A2. Pre- and Post-Treatment Outcomes Analyses for Treatment Engagers (N=240) with Complete Data**

|  | **Pre Mean (sd)** | **Post Mean (sd)** | **Statistic** | **Standardized Effect**  **Size [95% CI]** | **Between-Subject Variance (sd) and Residual Variance (sd)** |
| --- | --- | --- | --- | --- | --- |
| **Beck Depression Inventory (BDI-II) Total** | 21.06 (7.96) | 13.73 (8.99) | b=-7.33, SE=0.55, t(239)=-13.39, p < .001 | β=-0.79 [−0.91, -0.68] | σ²=36.15 (6.01)  σ²=35.96 (6.00) |
| **Patient Health Questionnaire (PHQ-9) Total** | 10.88 (5.46) | 8.00 (5.58) | b=-2.88, SE=0.30, t(236)=-9.50, p < .001 | β=-0.51 [−0.61, -0.40] | σ²=19.60 (4.43)  σ²=10.91 (3.30) |
| **Parkinson’s Anxiety Scale (PAS) Total** | 19.58 (9.36) | 14.34 (8.50) | b=-5.24, SE=0.50, t(236)=-10.46, p < .001 | β=-0.56 [−0.67, -0.46] | σ²=50.11 (7.08)  σ²=29.77 (5.46) |
| **PAS-Persistence Subscale** | 10.26 (4.43) | 7.60 (4.26) | b=-2.66, SE=0.25, t(236)=-10.59, p < .001 | β=-0.59 [−0.70, -0.48] | σ²=11.39 (3.37) σ²=7.49 (2.74) |
| **PAS-Episodic Subscale** | 5.00 (3.39) | 3.65 (2.86) | b=-1.35, SE=0.19, t(236)=-6.97, p < .001 | β=-0.42 [−0.54, -0.30] | σ²=5.37 (2.32)  σ²=4.45 (2.11) |
| **PAS-Avoidant Subscale** | 4.32 (3.00) | 3.10 (2.52) | b=-1.23, SE=0.18, t(236)=-6.86, p < .001 | β=-0.43 [−0.56, -0.31] | σ²=3.89 (1.97)  σ²=3.80 (1.95) |
| **UCLA 3-item Loneliness Scale (3LS) Total** | 5.45 (1.73) | 5.07 (1.62) | b=-0.37, SE=0.14, t(137)=-2.73, p=.007 | β=-0.22 [−0.38, -0.06] | σ²=1.53 (1.24)  σ²=1.26 (1.12) |

Linear Mixed-Effects Model of Pre-Post Symptom Scores with random effects for each subject. All effects are significant, however PAS and 3LS significance was only found in the CBT-PD Treatment group and not the MBCT-PD group (**Table 3**).

**Figure A1. Pre- and Post-Treatment Depression and Anxiety Scores for Treatment Engagers (N=240) with Complete Data**

**
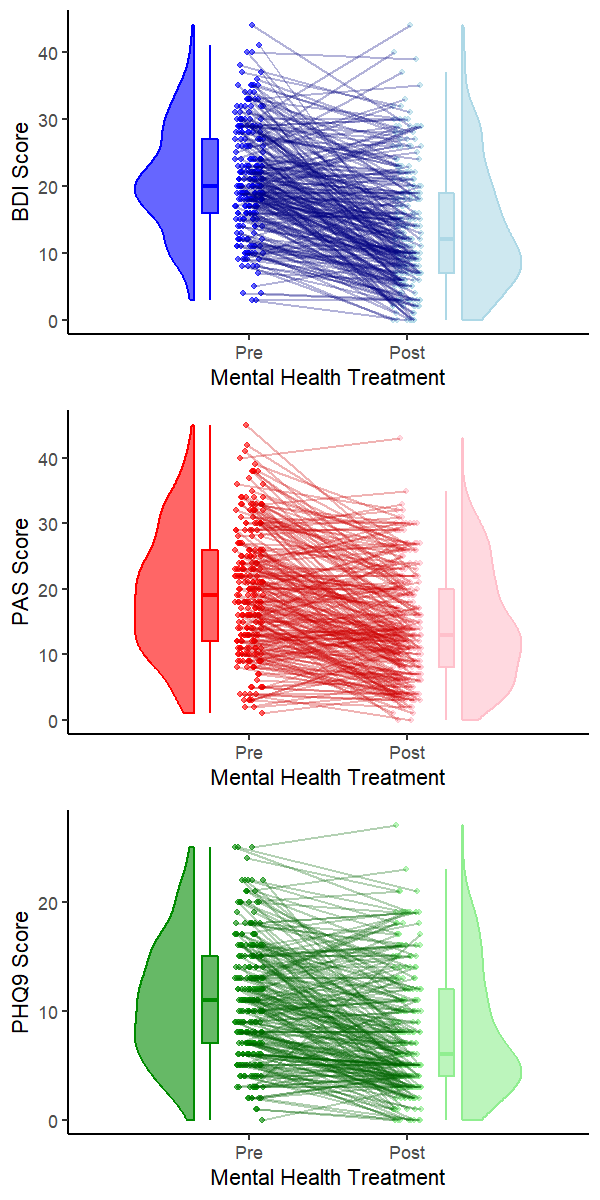
**
